# A framework to distinguish healthy/cancer renal CT images using the fused deep features

**DOI:** 10.3389/fpubh.2023.1109236

**Published:** 2023-01-30

**Authors:** Venkatesan Rajinikanth, P. M. Durai Raj Vincent, Kathiravan Srinivasan, G. Ananth Prabhu, Chuan-Yu Chang

**Affiliations:** ^1^Division of Research and Innovation, Department of Computer Science and Engineering, Saveetha School of Engineering, SIMATS, Chennai, Tamil Nadu, India; ^2^School of Information Technology and Engineering, Vellore Institute of Technology, Vellore, India; ^3^School of Computer Science and Engineering, Vellore Institute of Technology, Vellore, Tamil Nadu, India; ^4^Department of Computer Science Engineering, Sahyadri College of Engineering and Management, Mangaluru, India; ^5^Department of Computer Science and Information Engineering, National Yunlin University of Science and Technology, Yunlin, Taiwan; ^6^Service Systems Technology Center, Industrial Technology Research Institute, Hsinchu, Taiwan

**Keywords:** kidney cancer, renal CT slices, deep learning, KNN classifier, validation

## Abstract

**Introduction:**

Cancer happening rates in humankind are gradually rising due to a variety of reasons, and sensible detection and management are essential to decrease the disease rates. The kidney is one of the vital organs in human physiology, and cancer in the kidney is a medical emergency and needs accurate diagnosis and well-organized management.

**Methods:**

The proposed work aims to develop a framework to classify renal computed tomography (CT) images into healthy/cancer classes using pre-trained deep-learning schemes. To improve the detection accuracy, this work suggests a threshold filter-based pre-processing scheme, which helps in removing the artefact in the CT slices to achieve better detection. The various stages of this scheme involve: (i) Image collection, resizing, and artefact removal, (ii) Deep features extraction, (iii) Feature reduction and fusion, and (iv) Binary classification using five-fold cross-validation.

**Results and discussion:**

This experimental investigation is executed separately for: (i) CT slices with the artefact and (ii) CT slices without the artefact. As a result of the experimental outcome of this study, the K-Nearest Neighbor (KNN) classifier is able to achieve 100% detection accuracy by using the pre-processed CT slices. Therefore, this scheme can be considered for the purpose of examining clinical grade renal CT images, as it is clinically significant.

## 1. Introduction

It is becoming increasingly apparent that infectious and acute syndromes are rising worldwide. Appropriate clinical procedures are necessary for detecting and treating these diseases as early as possible. Untreated diseases will likely result in various problems, including death, and they may also burden the healthcare system substantially. It should be noted that acute diseases are usually more severe than infectious diseases. Compared with infectious diseases, acute diseases will also lead to death in individuals. According to the current literature, cancer is a severe acute disease that accounts for a substantial number of deaths worldwide and has been identified as a disease that causes many deaths as well (1–3).

A report published by the World Health Organization (WHO) in 2020 shows that cancer was the leading cause of death worldwide in 2020 and is expected to continue in that way.^1^ Several studies have indicated that, in the year 2020, approximately 10 million people will have died worldwide from various cancer-related causes, including cancer of the internal and external organs. According to this report, lung and colon cancer are the leading causes of death worldwide.

The Global Cancer Observatory (GCO) report lists several cancer cases in various body organs.^2^ This report lists cancer in organs based on its occurrence rate, cancer in the kidney is listed as the 14th most dangerous disease, and untreated renal cancer will lead to death. This report also confirms that, in 2020, the number of cancer patients increased to 431,288. This report also confirms that nearly 430,000 new cases will be diagnosed in 2020 alone. According to the disease prediction by GCO, kidney (renal) cancer is severe, and its occurrence rate is gradually rising due to various causes. Early recognition and management are compulsory to cure the disease completely using appropriate medications. Kidney cancer (KC) is commonly assessed by automatic methods using a chosen medical imaging dataset (renal CT images), and the achieved results are analyzed and recorded for further investigation.

The earlier studies in the literature confirm that renal CT (RCT)-based kidney detection is a recommended procedure to precisely detect kidney abnormality during the disease screening process. Usually, the RCT is collected as a three-dimensional (3D) image, and then, a 3D to 2D conversion is employed to reduce the computation complexity during the RCT analysis (4, 5). The axial-plane 2D slices are commonly adopted in the literature, and it helps to provide the necessary information about abdominal conditions, including kidney health. Hence, this study also considered the axial-plane 2D RCT slices to examine the KC. Before implementing the detection task, every image is resized to a recommended dimension.

The ultimate task of this investigation is to prepare a disease detection structure to accurately identify the KC using the RCT images with the help of the chosen deep-learning scheme. To achieve better detection accuracy, this study implemented a preprocessing image procedure to treat the raw renal CT using a threshold filter approach discussed in earlier research. In the earlier studies, this arrangement is considered to strip the skull region from the brain MRI slices (6, 7) and to remove the artifact in lung CT slices (8–10). A similar procedure is adopted in this study to remove the artifact in RCT slices to improve the visibility of the kidney section. The proposed cancer detection framework consists of the following phases:

i. Image resizing and artifact removal using threshold filter.
ii. Deep feature extraction using chosen pre-trained methods.
iii. Dual-deep feature generation using serially concatenated deep features.
iv. Binary classification and verification using a 5-fold cross-validation.

The merit of the computerized scheme depends on its explainability and robustness, and hence, this study considered a framework that is very simple and robust (11). This scheme considered MATLAB for initial image processing, and the developed framework is implemented using PYTHON. The experimental exploration is separately implemented using (i) RCT with the artifact and (ii) RCT without the artifact, and the achieved performance values are compared. This approves that the classification accuracy realized with the artifact-removed RCT is better than the raw RCT. Furthermore, this study employs pre-trained schemes, such as VGG16, VGG19, ResNet50, ResNet101, DenseNet121, and DenseNet201, to obtain better detection in the considered task. The results authorize that the outcome achieved with VGG19 and DenseNet121 is better for the chosen RCT, and hence, the proposed scheme is implemented using deep features of (i) VGG19 and (ii) DenseNet121 and serially concatenated features of VGG19 and DenseNet121 after a 50% dropout. The deep feature-based classification helps accomplish an accuracy of 100% with the RCT without the artifact. This confirms that the proposed framework is clinically noteworthy and can be considered to identify the KC from the RCT collected from actual patients.

The key contributions of this framework include the following:

i. Threshold filter-supported preprocessing is executed to eliminate artifacts in RCT.
ii. Implementation of the proven deep-learning schemes to detect the KC using RCT.
iii. Implementation of serially concatenated deep features to enhance the KC detection accuracy.

This study is divided into the following sections: Section 2 presents the context, Section 3 illustrates the methodology, and Sections 4 and 5 discuss the results and conclusions.

## 2. Related studies

Computerized disease screening and diagnosis is one of the recent advancements, adopted in a variety of hospitals and disease screening laboratories to reduce the diagnostic burden of doctors and lab technicians. The increased disease occurrence rates need a faster and more accurate system to detect the disease using chosen medical data. The bio-image-supported disease screening is a common and widely adopted procedure to verify the condition of the internal organs. Furthermore, the bio-image-supported methods support accurate disease information compared with other medical modalities, and hence, these methods are widely employed to screen patients suffering due to cancer.

Kidney cancer is one of the acute diseases and ranked 14th based on the year 2020 reports of the WHO and GCO. Appropriate diagnosis and treatment will help the patient to recover from the disease. Due to its importance, a number of computerized schemes are discussed by the researchers to distinguish the KC using RCT pictures. Table 1 summarizes a few chosen KC detection procedures found in the literature.

**Table 1:** Summary of the renal CT image examination methods.

**References**	**Procedure implemented**	**Outcome**
Alzu'bi et al. (12)	This study presents a new database of RCT images, which has been created using VGG16 code and ResNet50 support, which is used to detect the KC	97% accuracy
Xu et al. (13)	As a result of the implementation of ResNet50 and ResNet101, the cropped RCT images have been classified into the following two categories: healthy and cancerous	>82% accuracy
Amiri et a l. (14)	The execution of the machine learning scheme with a radiomics feature is discussed in order to detect kidney abnormalities by using RCT slices to perform the machine learning	94% accuracy
Miskin et al. (15)	With the application of machine learning techniques based on the cropped RCT images, the detection of benign and malignant cystic renal masses can be accomplished	93% specificity
Shehata et al. (16)	An innovative computer-assisted diagnosis system is proposed for examining kidney cancer in cropped RCT slices using a novel comprehensive renal cancer computer-assisted diagnosis scheme	89.6% accuracy
Nikpanah et al. (17)	A deep-learning-supported technique based on multi-phasic MRI is presented as an example of the execution of the technique for detecting the clear cell renal cell carcinoma	81% accuracy
Heller et al. (18)	With the use of the KiTS19 challenge benchmark 3D RCT images, we are able to segment the abnormal kidney region using 3D U-Net	Dice value of 97.4 and 0.85.1% is achieved for kidney and tumor, respectively
Bhandari et al. (19)	The present study discusses the detection of low/high-grade renal cell cancers from RCT images in detail	82 to 96% Area Under Curve (AUC) is present.
Islam et al. (20)	The work presented here implemented the VGG16, InceptionV3, and resNet50 using RCT slices to detect kidney abnormalities in multi-classes, with the VGG16 presenting a better detection metric compared to the others	98.2% accuracy

Along with the above-considered studies, the research by Abdelrahman and Viriri (21) presents a detailed survey on traditional and deep-learning segmentation of the abnormal fragment in the kidney in RCT images. The research by Wang et al. (22) also presents a thorough evaluation of the deep-learning-supported scheme for biomedical image examination, including the RCT. These studies authorize the need for a well-organized methodology to detect abnormality from the chosen medical image. Hence, in this research, a framework based on deep learning is proposed to detect the KC from the axial-plane RCT slices accurately.

## 3. Methodology

Using a binary classifier, this research division demonstrates how RCT slices are classified into healthy and cancerous classes in an axial plane. When the patient visits the nephrologist to verify the condition of the kidney, a recommended clinical protocol will be followed by the doctor to examine the kidney and its condition, and based on the observations/disease symptoms, the nephrologist recommends a bio-imaging-based examination to get the complete information about the kidney. When the patient undergoes a CT scan, it will provide a 3D picture of the abdominal region, which is then converted into 2D to reduce the computational complexity. Furthermore, the personal verification of the kidney section from the bio-image needs a 2D picture printed on a specialized film. A similar procedure is executed when a computer-supported diagnosis is implemented.

**Figure 1:**
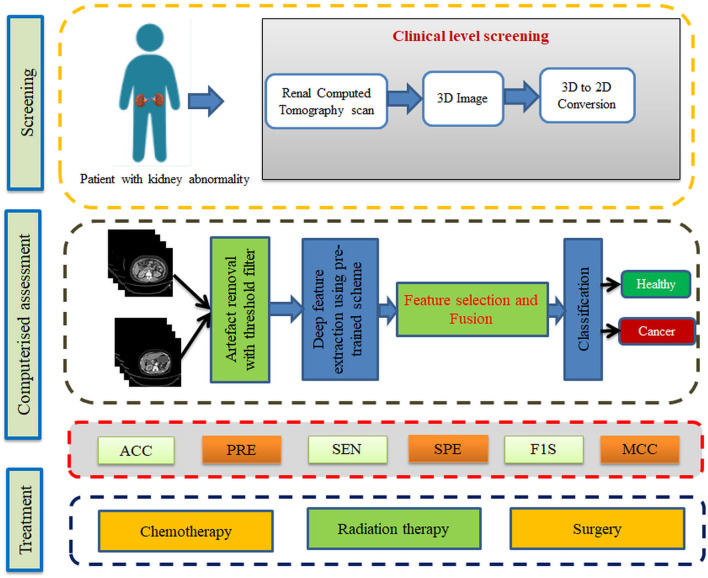
Kidney cancer detection framework.

From the data collection to the decision-making process, the proposed scheme is depicted in Figure 1. A number of procedures are involved in the proposed scheme, including image collection and preprocessing for improved detection accuracy, feature extraction utilizing a selected deep-learning technique, feature reduction, and serial feature concatenation to produce the fused feature vector, binary classification *via* 5-fold cross-validation, and verification of the proposed scheme's performance on the basis of the results obtained. In this study, the fused feature vector is constructed by integrating the deep features of VGG19 and DenseNet121. In addition, based on the computation of performance measures, the merit of the proposed scheme is confirmed based on the evaluation of these features to determine the classification performance of SoftMax and other binary classifiers.

### 3.1. Image database

**Figure 2:**
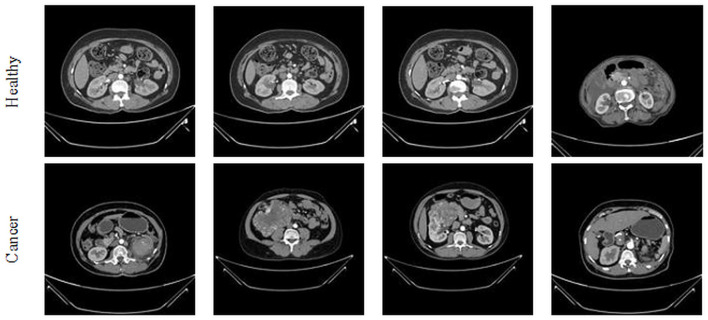
Sample axial-plane test images of renal CT slices.

This study considered the axial-plane RCT slices provided by Islam et al. (21). This dataset consists of both the axial-plane and coronal-plane images with categories, such as cyst, stone, cancer, and healthy. In this study, only the healthy and cancer axial-plane images alone are considered for the examination. To have a balanced database, this study considered 2,680 images (1,340 healthy class and 1,340 cancer class). Before implementing the classification task, every image is resized to 224 × 224 × 1 pixels. The proposed detection task is implemented using the RCT with and without the artifact, and the obtained results are separately examined and verified. Figure 2 represents the trial imageries considered in this study, and the number of images considered in this study is depicted in Table 2. In this study, 80% of images are considered to train the deep-learning scheme, 10% of images are considered for validation, and the remaining 10% of images are used to test the performance of the scheme with a 5-fold cross-validation with individual and fused features.

**Table 2:** Dataset considered to verify the proposed framework.

**Class**	**Dimension**	**Number of images**			

		**Total**	**Training (80%)**	**Validation (10%)**	**Testing (10%)**
Healthy	224 × 224 × 1	1,340	1,072	134	134
Cancer	224 × 224 × 1	1,340	1,072	134	134

#### 3.1.1. Artifact removal

**Figure 3:**
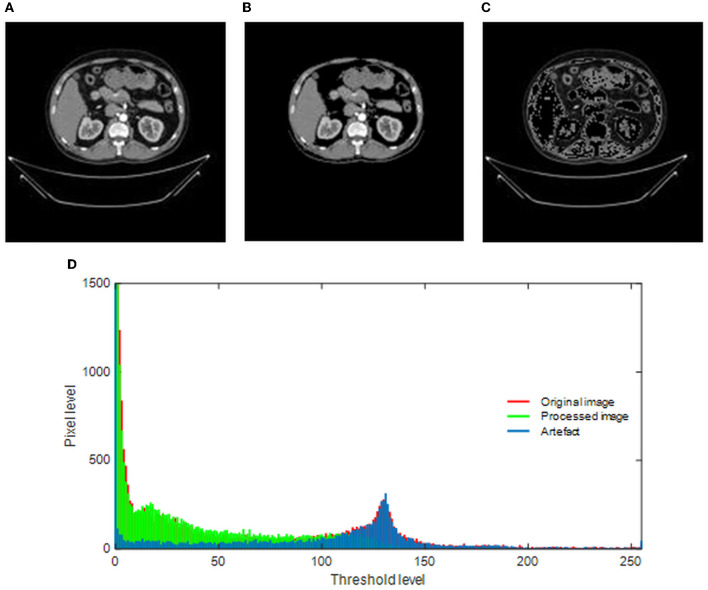
Implementation of threshold filter to eliminate the artifact. **(A)** Original image. **(B)** Processed image. **(C)** Artifact. **(D)** Gray-scale histogram.

The merit of the automatic medical image examination procedure depends mainly on the image database considered during the experimental investigation. The earlier studies in the research verify that the images without the artifact help in achieving a better accuracy compared with the images with the artifact (23). This study implements a threshold filter-supported method to remove the artifact from the chosen RCT, and this task is executed using MATLAB software as discussed in (24). In this process, the threshold value (Th), which separates the image into a processed artifact, is identified manually. When an appropriate Th is obtained, it is implemented to divide the raw test image into two sections as shown in Figure 3. Figure 3A shows the raw RCT, and Figures 3B, C shows the processed picture and the removed artifact. This task depends on the threshold level of the image, and it is shown in Figure 3D. The original histogram (red) depicts the pixel distribution of the raw RCT, the green histogram depicts the pixel distribution of the processed image, and the remaining section (blue) shows the pixel value of the artifact.

### 3.2. Deep-learning model

Recently, pre-trained and customized deep-learning procedures have been widely implemented in various data analytic tasks due to their performance, ease of implementation, and significance. Compared to the traditional and machine-learning schemes, the deep-learning procedures efficiently provide a better result on moderate and large datasets. Furthermore, most of these methods can be practically implementable in a chosen hardware system, improving its performance (25–27).

Researchers have recently widely employed pre-trained models to achieve better results during medical image examination tasks. The proposed research study also implements well-known pre-training procedures, such as VGG16, VGG19, ResNet50, ResNet101, DenseNet121, and DenseNet201, to examine the KC in RCT slices. The complete evidence concerning the preferred schemes can be found in the literature (28–32), and in this study, these schemes are considered along with chosen binary classifiers. The following initial parameters are assigned for these models: learning rate = 1 × 10^−5^, training with linear dropout rate (LDR), Adam optimization, ReLu activation, total iteration = 2000, total epochs = 150, and classification with a SoftMax unit using a 5-fold cross-validation.

Before implementing the developed scheme, an image augmentation procedure is implemented to increase the learning capability of the chosen deep-learning systems. The augmentation process involves the horizontal and vertical flip, an angle-based rotation, and zoom-in and zoom-out. This helps the system to learn better about the features of the image.

### 3.3. Feature vector generation and classification

Each deep-learning procedure implemented in this study provides a deep feature vector of dimension 1 x 1 x 1,000, which is then used to authenticate the merit of the classifiers. The feature vector after a 50% dropout will offer a reduced feature vector of dimension 1 x 1 x 500, which is the concatenated deep feature with similar reduced features to achieve a fused feature vector of dimension 1 x 1 x 1,000, which helps in achieving a better classification accuracy during the RCT-based KC detection task. The total dimension of these features is 1 x 1 x 1,000, which is then reduced to 1 x 1 x 500 using a 50% dropout, and from this, the fused feature vector is obtained. The feature vectors of this system are depicted in Equations (1)–(3) (33, 34):


(1) $$DLF_{VGG19\;(1\times 1\times 1000)}=VGG19_{1,1},VGG19_{1,2},...,VGG19_{\left(1,1000\right)}$$

(2) $$\begin{array}{lll} DLF_{DenseNet121\;\left(1\times 1\times 1000\right)} & = & DN_{1,1},DN_{1,2},...,DN_{\left(1,1000\right)} \end{array}$$

(3) $$\begin{array}{l} DLF_{VGG+DN\;(1\times 1\times 1000)}=\;VGG+DN_{1,1},VGG\\ \;+\;DN_{1,2},...,VGG+DN_{\left(1,1000\right)} \end{array}$$

where DLF = deep-learning features, VGG = VGG19, and DN = DenseNet121.

### 3.4. Performance metric computation

Performance metrics obtained during the classification task are used to verify the merit of the proposed scheme. To begin with, the measures, such as true-positive (TP), false-positive (FP), true-negative (TN), and false-negative (FN), are computed from the confusion matrix presented in Equations, which are then used to implement these values into mathematical expressions. From Equations (4) to (9), the necessary measures, such as accuracy (ACC), precision (PRE), sensitivity (SEN), specificity (SPE), F1-score (F1S), and Matthews correlation coefficient (MCC), are calculated. In contrast to the binary classification task in this study, SoftMax, Nave-Bayes (NB), decision trees (DT), random forests (RF), KNNs, and support vector machine (SVM) are used (35–37).


(4) $$\begin{array}{lll} ACC & = & \frac{TP+TN}{TP+TN+FP+FN}\; \end{array}$$

(5) $$\begin{array}{lll} PRE & = & \frac{TP}{TP+FP}\; \end{array}$$

(6) $$\begin{array}{lll} SEN & = & \frac{TP}{TP+FN}\; \end{array}$$

(7) $$\begin{array}{lll} SPE & = & \frac{TN}{TN+FP}\; \end{array}$$

(8) $$\begin{array}{lll} F1S & = & \frac{2TP}{2TP+FP+FN}\; \end{array}$$

(9) $$\begin{array}{lll} MCC & = & \frac{\left(TP*TN\right)-\left(FP*FN\right)}{\sqrt{\left(TP+FP\right)*\left(TP+FN\right)*\left(TN+FP\right)*\left(TN+FN\right)}} \end{array}$$

## 4. Results and discussions

The proposed study is implemented with MATLAB and Python on a workstation equipped with an Intel i7 2.9 GHz processor, 20 GB RAM, and 4 GB VRAM.

Initially, the proposed framework is implemented on the raw RCT images with the artifacts, and the classification performance is verified using the chosen binary classifiers. Then, the RCT classification performance of chosen pre-trained models is verified using the raw axial-plane images, and the outcomes are equated. The outcome of this experiment authorizes that the SoftMax-based binary classification with a 5-fold cross-validation provides a better detection performance with VGG19 and DenseNet121 methods compared with VGG16, ResNet50, ResNet101, and DenseNet201. Furthermore, along with the detection accuracy, the MCC achieved with these schemes is also better; this information is shown in Table 2.

**Figure 4:**
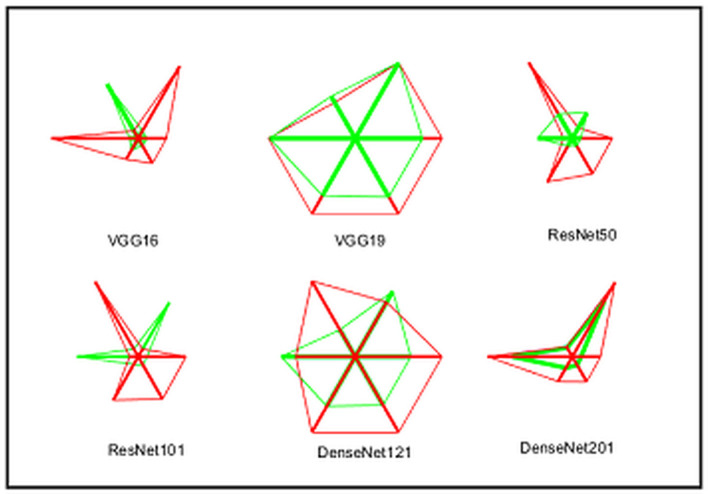
Integrated Glyph plot to demonstrate the overall performance of the considered methods.

A similar experimental task is repeated using the images whose artifacts are eliminated with a threshold filter. The results of this study confirm that this process offers a better ACC and MCC than other methods, as represented in Table 3. This table also approves that the VGG19 and DenseNet121 offer better performance. **Table 5** presents the outcome for VGG16 with a SoftMax for various folds, and the best fold value is chosen as the outcome. The result of a chosen cross-validation approach is also presented in Figure 4. In this figure, the Glyph plot of Tables 3, 4 is separately developed and merged. These images are necessary to confirm the overall merit of this scheme, and this confirms that the artifact-removed RCT provides a better result than other methods. In addition, the result authorizes that this structure works fine on the chosen RCT images.

**Table 3:** Classification results achieved for raw renal CT slice with a SoftMax classifier.

**Scheme**	**TP**	**FN**	**TN**	**FP**	**ACC**	**PRE**	**SEN**	**SPE**	**F1S**	**MCC**
VGG16	118	15	116	19	87.3134	86.1314	88.7218	85.9259	87.4074	74.6644
VGG19	118	17	121	12	**89.1791**	**90.7692**	**87.4074**	**90.9774**	**89.0566**	**78.4176**
ResNet50	120	13	116	19	88.0597	86.3309	90.2256	85.9259	88.2353	76.2024
ResNet101	117	20	117	14	87.3134	89.3130	85.4015	89.3130	87.3134	74.7144
DenseNet121	118	18	119	13	**88.4328**	**90.0763**	**86.7647**	**90.1515**	**88.3895**	**76.9269**
DenseNet201	118	20	116	14	87.3134	89.3939	85.5072	89.2308	87.4074	74.7130

The bold contents are the considered best metric.

**Table 4:** Classification results achieved for processed renal CT slice with a SoftMax classifier.

**Scheme**	**TP**	**FN**	**TN**	**FP**	**ACC**	**PRE**	**SEN**	**SPE**	**F1S**	**MCC**
VGG16	124	9	129	6	94.4030	95.3846	93.2331	95.5556	94.2966	88.8257
VGG19	127	7	128	6	**95.1493**	**95.4887**	**94.7761**	**95.5224**	**95.1311**	**90.3010**
ResNet50	125	8	127	8	94.0299	93.9850	93.9850	94.0741	93.9850	88.0590
ResNet101	128	5	126	9	94.7761	93.4307	96.2406	93.3333	94.8148	89.5939
DenseNet121	129	5	127	7	**95.5224**	**94.8529**	**96.2687**	**94.7761**	**95.5556**	**91.0549**
DenseNet201	126	9	127	6	94.4030	95.4545	93.3333	95.4887	94.3820	88.8295

The bold contents are the considered best metric.

**Figure 5:**
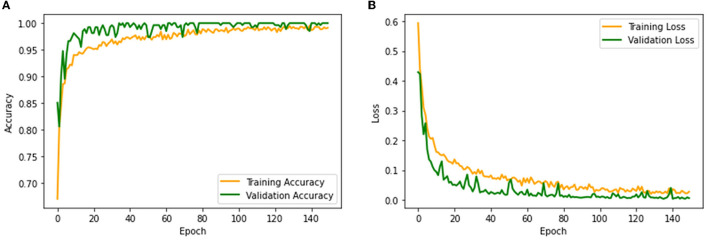
Convergence of training and validation process. **(A)** Accuracy. **(B)** Loss.

**Figure 6:**
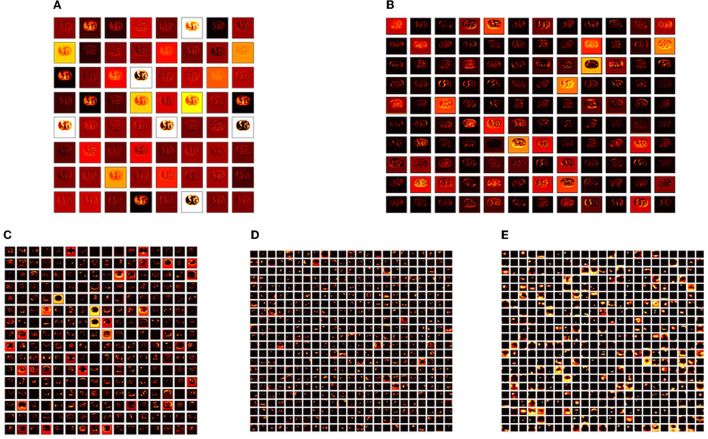
Intermediate layer outcomes collected from VGG19. **(A)** Conv1. **(B)** Conv2. **(C)** Conv3. **(D)** Conv4. **(E)** Conv5.

**Figure 7:**
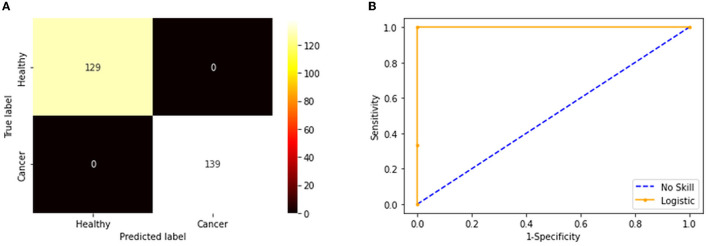
Confusion matrix and ROC curve achieved with fused features. **(A)** Confusion matrix. **(B)** the ROC curve.

The performance of the proposed system is then verified by considering the fused deep features of dimension 1 x 1 x 1,000. During this task, the VGG19 and DenseNet121 features are considered. Then, their features are sorted based on their value, and finally, a 50% dropout of these features is employed. To execute the classification task, the attained features are then serially fused to achieve a fused feature vector with dimensions of 1 x 1 x 1,000 pixels. The result of this experiment with fused features is presented in Figures 5–7. Figure 5 presents the convergence achieved with RCT image databases, and this figure confirms that the proposed method helps to achieve better detection accuracy (1,000%) than other methods. Figures 5A, B denote the experimental result achieved in this study.

The convolutional layer outcome was extracted with these results to verify the framework's performance with the chosen database. The results of Figure 6 show that this method will provide a better result and efficiency in completing the task. Figure 7 presents the outcome of the proposed technique, which shows the confusion matrix and the receiver operating characteristic (ROC), which depends mainly on the test images considered. The ROC value achieved is improved compared with the alternatives.

The result of this method authorizes that this system benefits in achieving a better result, and these measures for the experiment with conventional and fused features are shown in Table 5. The initial result for this table is achieved using a VGG19 and DenseNet121, which confirms the merit of the proposed technique. Finally, a spider plot is constructed to demonstrate the result in a graphical form, and the best result is highlighted.

**Table 5:** Outcome of VGG16 with SoftMax for a 5-fold cross-validation.

**Cross-validation**	**TP**	**FN**	**TN**	**FP**	**ACC**	**PRE**	**SEN**	**SPE**	**F1S**	**MCC**
Fold 1	125	10	128	5	94.4030	96.1538	92.5926	96.2406	94.3396	88.8703
Fold 2	126	7	126	9	94.0299	93.3333	94.7368	93.3333	94.0299	88.0702
Fold 3	121	11	130	6	93.6567	95.2756	91.6667	95.5882	93.4363	87.3645
Fold 4	127	5	126	10	94.4030	92.7007	96.2121	92.6471	94.4238	88.8716
Fold 5	127	7	128	6	**95.1493**	**95.4887**	**94.7761**	**95.5224**	**95.1311**	**90.3010**

The bold contents are the considered best metric.

**Figure 8:**
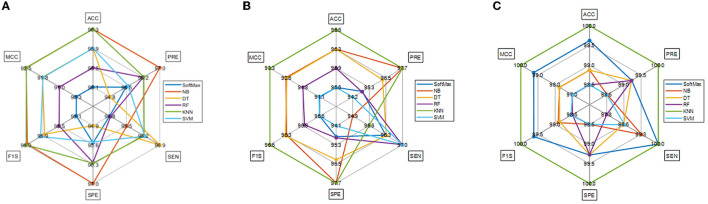
Spider plot achieved using the results of Table 6. **(A)** VGG19. **(B)** DenseNet121. **(C)** Fused deep features (VGG+DN).

The task of the proposed scheme is successfully employed using the fused features, and this scheme helps to accomplish an improved recognition accuracy (100%) compared with other methods found in the literature. The performance evaluation of Table 6 presented in Figure 8 confirms its overall merit on various classifiers. Figures 8A–C present the classification performance for different feature vectors. The main limitation of this research is the implementation of the threshold filter, which needs a manually verified Th. Nevertheless, the merit of the proposed scheme is verified using the clinical grade CT database, and the achieved experimental outcome verifies that the planned technique is better and helps to get better detection accuracy. The limitation of the proposed study is it needs an artifact removal process and it can be replaced by a chosen image enhancement scheme to achieve better disease detection accuracy.

**Table 6:** Overall results achieved with the proposed framework for individual and fused features.

**Image**	**Classifier**	**TP**	**FN**	**TN**	**FP**	**ACC**	**PRE**	**SEN**	**SPE**	**F1S**	**MCC**
VGG19	SoftMax	127	7	128	6	95.1493	95.4887	94.7761	95.5224	95.1311	90.3010
		NB	128	6	130	4	**96.2687**	**96.9697**	**95.5224**	**97.0149**	**96.2406**	**92.5476**
		DT	127	4	130	7	95.8955	94.7761	96.9466	94.8905	95.8491	91.8141
		RF	128	7	128	5	95.5224	96.2406	94.8148	96.2406	95.5224	91.0554
		KNN	129	5	129	5	96.2687	96.2687	96.2687	96.2687	96.2687	92.5373
		SVM	130	5	127	6	95.8955	95.5882	96.2963	95.4887	95.9410	91.7927
DenseNet121	SoftMax	129	5	127	7	95.5224	94.8529	96.2687	94.7761	95.5556	91.0549
		NB	129	7	129	3	96.2687	97.7273	94.8529	97.7273	96.2687	92.5802
		DT	130	5	128	5	96.2687	96.2963	96.2963	96.2406	96.2963	92.5369
		RF	128	4	129	7	95.8955	94.8148	96.9697	94.8529	95.8801	91.8150
		KNN	129	6	130	3	**96.6418**	**97.7273**	**95.5556**	**97.7444**	**96.6292**	**93.3077**
		SVM	129	4	127	8	95.5224	94.1606	96.9925	94.0741	95.5556	91.0868
Fused deep features (VGG+DN)	SoftMax	133	0	134	1	99.6269	99.2537	100	99.2593	99.6255	99.2565
		NB	137	1	128	2	98.8806	98.5612	99.2754	98.4615	98.9170	97.7614
		DT	132	2	133	1	98.8806	99.2481	98.5075	99.2537	98.8764	97.7639
		RF	132	3	132	1	98.5075	99.2481	97.7778	99.2481	98.5075	97.0259
		KNN	139	0	129	0	**100**	**100**	**100**	**100**	**100**	**100**
		SVM	136	2	128	2	98.5075	98.5507	98.5507	98.4615	98.5507	97.0123

The bold contents are the considered best metric.

## 5. Conclusion

The literature authorizes that cancer is a severe disease in human communities, and early diagnosis and treatment are necessary. When the cancer is accurately diagnosed, it can be controlled using a recommended clinical protocol. Due to its importance, a substantial amount of automatic cancer detection based on the bio-image-supported technique has been proposed and executed by researchers (38). The proposed study aims to develop a framework to effectively detect the KC in RCT images with the help of pre-trained deep-learning procedures. This study considered VGG19 and DenseNet121 schemes to classify the RCT into healthy/cancer classes with improved accuracy. As part of this study, individual DLFs and fused DLFs are employed to perform the binary classification task, and the results are compared to identify the most appropriate KC scheme. According to the results of this study, a binary classification with a KNN classifier was effective in achieving an accuracy of 100% for RCTs that had previously been preprocessed using a threshold filter. Based on the results of this research, the proposed framework appears to be effective, and it will be possible to test and validate its performance using clinically collected RCT slices in future.

## Data availability statement

The original contributions presented in the study are included in the article/supplementary material, further inquiries can be directed to the corresponding author.

## Author contributions

KS and C-YC conceptualized and supervised the research and carried out the project administration and validated the results. VR contributed to the development of the model, data processing, training procedures, and implementation of the model. VR, PV, and KS wrote the manuscript. VR, PV, KS, GA, and C-YC reviewed and edited the manuscript. C-YC carried out the funding acquisition. All authors contributed to the article and approved the submitted version.

## Footnotes

^1^ https://www.who.int/health-topics/cancer

^2^ https://gco.iarc.fr/
